# Halting COVID-19 Requires Collective, Decentralized, and Community-Led Responses

**DOI:** 10.1007/s42413-022-00171-9

**Published:** 2022-06-09

**Authors:** Sudip Bhandari, Shadrack Osei Frimpong, Priya Darshini Bhirgoo

**Affiliations:** 1 Department of International Health, Johns Hopkins Bloomberg School of Public Health, Baltimore, MD USA; 2 Yale School of Public Health, 464 Congress Avenue, New Haven, CT 06520 USA; 3 University of Cambridge, Cambridge, UK; 4 Cocoa360, Accra, Ghana; 5 University of Pennsylvania, Philadelphia, PA USA

**Keywords:** COVID-19, Global health, Paradigm shift, Governance, Regional coordination, Community involvement

## Abstract

Many global health organizations are reliant on the funding provided by a few dozen high-income countries, making them fiscally insecure and fragile, especially during times of global crises. The COVID-19 pandemic could be an opportunity to move away from this status quo to a more decentralized, multipolar, and community-led approach. The global health community can take four immediate steps in response to the pandemic to start that paradigm shift now: support more regional and country-specific responses, convince national and regional business houses and philanthropies to make up for response funding shortfalls, leverage public health advocacy to improve investments in public health infrastructure, and put community leaders and members at the frontlines of mitigation efforts.

## Background

The severe acute respiratory syndrome coronavirus 2 (SARS-CoV-2), the virus that causes COVID-19, has spread globally since it first emerged in Wuhan, China, in December 2019. Despite the release of vaccines, many governments in low- and middle-income countries (LMICs) have been relying on public health measures such as community containment, quarantine, and social distancing to flatten the epidemic curve due to inadequate financing to implement mass vaccinations (Lawal et al., 2022). While some of these public health measures have helped slow the spread of the virus in a few countries and regions such as Taiwan, New Zealand, and Mauritius (Baker et al., 2020; Chan Sun & Lan Cheong Wah, 2020; Wang et al., 2020), the spread remains unabated in almost all other countries (Johns Hopkins University & Medicine, 2020). The repercussions have been enormous – hospitalization rates have soared, unemployment rates continue to worsen, and many communities' overall social fabric and well-being have been disrupted (Sibley et al., 2020).

Global health institutions like the World Health Organization (WHO), tasked with leading the response to this and other pandemics, face chronic budget constraints, limiting their response scope (Gostin, 2020). Most of their funding comes from a few dozen high-income countries (HICs). Historically, global health has been centered in the United States and the United Kingdom. As evidenced by the recent Global Health 50/50 report (2020), 85% of global health organizations have their headquarters in North America and Europe, and more than 80% of global health leaders are nationals of HICs (University College London, 2020). Unfortunately, the dependence of global health organizations on a handful of HICs makes them fiscally insecure and fragile, especially during times of global crisis when HICs are experiencing economic contractions of their own.

While the fragility of global health institutions is a challenge that needs to be overcome, COVID-19 also presents an opportunity to reflect on how global health financing efforts and public health responses can be reshaped around the world. As a global health community, there are four steps that we can take to move away from the global health status quo to a democratic, multipolar, and community-led approach that will allow communities around the world to fight against the COVID-19 pandemic more effectively.

## Four Steps Towards a Paradigm Shift in Global Health

First, the global health community should focus on supporting regional and country-specific responses. While high-income countries are struggling with their responses and a global economic recession is underway, low- and middle-income countries (LMICs) will need to fend for themselves. So, it is paramount that LMICs form regional alliances to monitor the spread of the virus, and share data, funding, and best practices. An example of such cooperation came in mid-March when eight South Asian nations—Afghanistan, Bangladesh, Bhutan, India, the Maldives, Nepal, Pakistan, and Sri Lanka—revived their almost defunct regional institution (SAARC) to accumulate funding for their fight against COVID-19 (Fruman & Kaul, 2020). There are bright spots from local efforts as well. Despite its modest economic status, India's Kerala state is inching towards flattening its epidemic curve through contact tracing and social assistance (Faleiro, 2020). Similarly, Sub-Saharan African countries, through the Africa Center for Diseases Control and Prevention (Africa CDC), have initiated mitigation efforts such as laboratory diagnosis, surveillance, screening at major airports and naval entry points, and the supply of Personal Protective Equipment (Paintsil, 2020). They have collaborated to leverage lessons from the Ebola Virus Disease (EVD) crisis, which claimed thousands of lives, primarily in Liberia, Guinea, Sierra Leone, and Nigeria. These collaborative efforts have aided the implementation of interventions suitable for these countries' unique economic strengths and population dynamics.

Secondly, national and regional business houses and philanthropies should step up to fill the shortage of COVID-19 response funding. While the Gates Foundation quickly committed USD 150 million after the United States decided to halt WHO funding (Wise, 2020), it is not enough. Global health non-profits and non-governmental organizations (NGOs) that operate in developing countries should marshal their efforts to fight against COVID-19 by supporting country-specific governments financially or otherwise. During the EVD epidemic, non-profit organizations such as Alliance for International Medical Action (ALIMA) and private entities such as Firestone provided Ebola Treatment Units (ETUs) and worked alongside community members to conduct contact tracing and provide health education (Kirsch et al., 2017; Martín et al., 2016). For a pandemic of magnitude as large as COVID-19, the collective efforts of all global health actors, ranging from funders to program-delivery organizations and their private allies, are needed more than ever.

A third approach is public health advocacy. With an overarching goal of changing pertinent upstream factors like regulations, laws, and institutional practices, public health advocacy has historically contributed significantly to public health promotion, including successful HIV/AIDS control and injury prevention campaigns (Chapman, 2001; Hubinette et al., 2017). However, existing evidence shows that, in LMICs such as Nigeria and South Africa, governments have historically underfunded public health priorities such as Health Policy and Systems Research (HPSR) and mental health care during the COVID-19 pandemic (Okedo-Alex et al., 2021; Davies et al., 2022). Advocacy mechanisms such as the print and electronic media and community coalitions could be engaged to rally governments and their private-sector allies to prioritize and amplify their investments in public health infrastructure and research.

Finally, and most importantly, communities should be actively engaged in the fight against the COVID-19 pandemic. A crucial lesson from the EVD crisis was the power of community involvement for an effective response. A significant portion of the flattening of the EVD epidemic curve was attributed to crucial behavioral changes at the community level rather than international efforts such as those of the United Nations Mission for Ebola Emergency Response, which intervened later (Martín et al., 2016). In the same vein, the spread of COVID-19 could also be slowed and eventually halted through interventions that empower community leaders and members to be at the forefront of contact tracing, quarantine and social distancing, and educational campaigns. Not only would community engagement help to stop the pandemic, but it could also prevent social resistance and retaliation, which can hamper mitigation efforts and further worsen the scale of the pandemic.

## Conclusions

When this pandemic subsides, the global community will still need a profound rethinking of the structures it wants to set for global health going forward. That should entail a reconsideration of funding mechanisms and an exploration of ways global health organizations can be strengthened, especially during crises. Such changes require public health advocacy and active involvement of regional, national, and community-based leaders who have historically been instrumental in pandemic responses. More importantly, the COVID-19 pandemic may be a critical juncture to translate the rhetoric of global health decentralization into action. The recommendations herein are immediate steps we can take to start that paradigm shift now.

## Data Availability

Not applicable.

## Abbreviations

CDC: Centers for Disease Control and Prevention
COVID: Corona Virus Disease
ETU: Ebola Treatment Unit
EVD: Ebola Virus Disease
HPSR: Health Policy and Systems Research
LMICs: Low- and Middle-Income Countries
NGO: Non-Governmental Organization
PPE: Personal Protective Equipment
SAARC: South Asian Association for Regional Cooperation
WHO: World Health Organization
